# Real-Life Metal Cocktail Induced Pancreatic Alterations in Rats: Influence of Sex and Exposure Duration

**DOI:** 10.3390/ijms27104624

**Published:** 2026-05-21

**Authors:** Katarina Baralić, Đurđica Marić, Zorica Bulat, Danijela Đukić-Ćosić, Ivan Milošević, Anita Radovanović, Tijana Lužajić Božinovski, Vera Lukić, Aleksandra Repić, Biljana Antonijević, Aleksandra Buha Djordjevic

**Affiliations:** 1 Department of Toxicology “Akademik Danilo Soldatović”, Faculty of Pharmacy, University of Belgrade, Vojvode Stepe 450, 11221 Belgrade, Serbia; djurdjica.maric@pharmacy.bg.ac.rs (Đ.M.); zorica.bulat@pharmacy.bg.ac.rs (Z.B.); danijela.djukic.cosic@pharmacy.bg.ac.rs (D.Đ.-Ć.); biljana.antonijevic@pharmacy.bg.ac.rs (B.A.); aleksandra.buha@pharmacy.bg.ac.rs (A.B.D.); 2 Department of Histology and Embryology, Faculty of Veterinary Medicine, University of Belgrade, Bulevar Oslobođenja 18, 11000 Belgrade, Serbia; ikavet@vet.bg.ac.rs (I.M.); anita@vet.bg.ac.rs (A.R.); ticavet@vet.bg.ac.rs (T.L.B.); 3 Institute of Forensic Medicine, Faculty of Medicine, University of Belgrade, Deligradska 31A, 11000 Belgrade, Serbia; vera_lukic2006@yahoo.com (V.L.); aleksandrarepic@gmail.com (A.R.)

**Keywords:** mixture toxicology, pancreatic function, redox status, sex-specific toxicity, metabolic disruption

## Abstract

Toxic metals from industrialization and urbanization pose major human health risks, and mixture-based exposure requires broader toxicity assessment. This study investigated the effects of a mixture of arsenic, lead, mercury, cadmium, chromium (VI), and nickel on pancreatic function in rats (45 male/45 female; *n* = 5 per group), focusing on sex- and duration-specific differences after 28 and 90 days of exposure. The metals were administered as a single mixture dissolved in deionised water via oral gavage. Evaluated parameters included pancreatic metal levels, histopathology, serum glucose, amylase, malate dehydrogenase 1 (MDH-1) activity, redox status, and bioelements. Dose levels were based on human exposure data to reflect realistic scenarios. Metals accumulated in pancreatic tissue, causing dose- and time-dependent histopathological changes, including acinar cell vacuolization, vascular congestion, and Langerhans islet alterations. Males showed more pronounced vascular and islet changes, while females had greater acinar alterations. In males, higher doses decreased glucose and amylase and increased MDH-1 activity, while females showed more variable responses. Males demonstrated adaptive responses to oxidative stress over time, while females experienced more persistent stress. These findings reveal sex-, dose-, and duration-dependent effects of toxic metal(oid) mixtures on pancreatic function, indicating that individually safe doses may be harmful when combined.

## 1. Introduction

Toxic metals are ubiquitous in the environment, originating from both natural processes and human activities. However, with the rapid pace of industrialization and urbanization, the release of these substances into the environment has significantly increased [1,2]. Human exposure to metals from industrial sources often follows a cycle: from industry to the air, soil, and water, eventually reaching people through their food and drinking water. Among these, food and water are the main sources of metal exposure for the general population [3]. The presence of toxic metals in the environment raises serious ecological and health concerns because of their potential long-term impacts on human health [4]. Recognizing the importance of this issue has prompted numerous studies on the harmful effects of these substances on human health. However, current risk assessments primarily concentrate on individual metals [5]. In real-world scenarios, people are often exposed to multiple toxic metals at once, indicating that assessing the toxicity of metal mixtures based only on individual components may be insufficient [6]. Metals of great importance to public health due to their toxicity and prevalence in the environment include cadmium (Cd), arsenic (As), mercury (Hg), lead (Pb), and chromium (Cr) [7]. Metals exert their harmful effects by affecting cell membranes, mitochondria, lysosomes, and DNA, as well as disrupting the functions of enzymes involved in various biological processes [8], while one of the primary mechanisms of their toxicity is the generation of reactive oxygen species (ROS) and their subsequent effect on the body’s antioxidant defences [7]. It has also been demonstrated that Cd, Pb, Hg, As, and nickel (Ni) can act as endocrine-disrupting chemicals (EDCs) [9,10]. The World Health Organization (WHO) defined EDCs as “exogenous substances or mixtures that alter the functions of the endocrine system and consequently cause adverse health effects in an intact organism, its offspring, or (sub)populations” [11]. According to National Institutes of Health (NIH), EDCs are “chemicals that mimic, block, or interfere with hormones in the body’s endocrine system” [12], while the European Commission defines EDCs as “chemicals which under certain conditions can impact on the hormonal system of humans and animals” [13]. Conventional toxicological tests, which are tightly controlled, often fail to accurately represent real-life exposure scenarios for EDCs. A potential solution lies in the design of long-term experiments using very low doses, a crucial approach in EDC research. Even small doses of these substances have been shown to affect endocrine function, and some EDCs may exhibit a non-monotonic dose–response, further complicating the interpretation of their effects [14,15]. Hence, with current knowledge, scientific and regulatory risk assessments now emphasize the toxicity of low doses of metal mixtures, reflecting the real-life exposures experienced by a significant portion of the population [16]. Endocrine disruptors impact metabolism and cellular function, causing imbalances in various systems, including disruptions in insulin production [17]. In addition to genetic and lifestyle factors (such as sedentary behaviour and unhealthy eating habits), environmental factors also significantly contribute to the development of type 2 diabetes [18,19]. Furthermore, over the past two decades, EDCs have become increasingly significant in the progression of this disease [20,21]. Pb, Cd and Hg have been found to notably disrupt mitochondrial energy processes in pancreatic beta cells. These disruptions include reduced ATP synthesis, impaired insulin release in response to glucose, and alterations in mitochondrial membrane potential, all of which are essential for proper pancreatic function. Among these metals, Cd emerged as the most toxic, severely impacting these cellular functions [22]. Metals like cadmium (Cd), Pb and Hg have been shown to lower the activity of essential pancreatic enzymes, such as amylase, trypsin, protease, and lipase, while this decline indicates a direct toxic impact on the pancreas [23]. Human epidemiological studies have identified a statistical association between the development of type 2 diabetes and long-term exposure to low levels of endocrine disruptors, including toxic metals [24]. Toxic metals accumulating in the human body are believed to impair β-cell function and survival, as well as disrupt insulin signalling in fat and muscle cells, thereby hindering glucose uptake [25]. Our previous research has found that the median blood levels of Cd, As, Hg, Cr, Ni, and Pb in women and men in non-occupationally exposed populations from Serbia who participated in the study were as follows: Cd (µg/L), 1.96 in women and 1.48 in men; As (µg/L), 10.44 in women and 0.97 in men; Hg (µg/L), 3.44 in women and 5.22 in men; Cr (µg/L), 1.06 in women and 1.32 in men; Ni (µg/L), 8.28 in women and 7.61 in men; and Pb (µg/L), 22.528 in women and 3.495 in men [26,27,28]. The same research has also revealed that blood and serum insulin levels were linked to concentrations of Cd, Hg, Cr, Ni, and As, with As showing the strongest correlation. We found a strong positive correlation between insulin levels and both Hg and As, while Ni was negatively correlated. Notably, these correlations were observed exclusively in women [29]. Likewise, we observed a positive link between Pb and serum insulin levels, with an established dose–response relationship [27].

Considering all of the above, we hypothesized that chronic low-dose exposure to a real-life mixture of toxic metals induces sex- and exposure-duration-dependent alterations in pancreatic function in rats, leading to metal accumulation that disrupts pancreatic tissue, structural damage, and impaired biochemical markers of exocrine and endocrine activity, with more pronounced effects after prolonged exposure. Hence, the current study aimed to investigate the impact of toxic metal(oid) (arsenic, lead, mercury, cadmium, chromium (VI), and nickel) mixtures on pancreatic function in rats, focusing on sex- and duration-specific differences during 28- and 90-day exposures.

## 2. Results

### 2.1. Toxic Metals

Toxic metal levels measured in pancreatic tissue are presented in Table 1. After 28 days of exposure, male rats exhibited elevated Pb levels across all the treated groups compared to the control, with the lowest increase observed in group M1. Cd levels were elevated in groups M2, M3, and M4 in comparison with the control, with the highest increase in group M4, which was also the case for Hg levels. As levels showed a significant rise only in groups M3 and M4, while Cr levels increased exclusively in group M4 when compared with the controls. Interestingly, Ni levels increased only in group M2 in comparison with the control. In female rats, Pb levels showed an increase exclusively in group F3 compared to the control. Similarly, As, Cr, and Ni levels were elevated only in the highest concentration group, F4, in comparison with the control. Cd levels increased in groups F2, F3, and F4 compared to the control, with the highest concentrations in F4. Additionally, Hg levels were elevated in groups F3 and F4. In male animals, after a 90-day exposure, Pb levels were elevated in groups M1, M2, and M3 compared to the control, while As and Ni levels were increased exclusively in group M3. Both Cd and Hg levels were increased in groups M2 and M3 compared to the control. Notably, there was no statistically significant change in Cr levels in any of the treated groups in comparison with the control. In female rats after a 90-day exposure, Pb, As and Cr levels were elevated in all treated groups (F1, F2, and F3), indicating a consistent accumulation across all the exposure levels. In contrast, Cd, Ni, and Hg levels were elevated only in the higher concentration groups (F2 and F3). In summary, after 28 days of exposure, male rats showed broader metal accumulation across the groups, while females exhibited increases mainly at higher concentrations or in specific groups. On the other hand, after 90 days, males showed group-specific metal increases, while in females consistent Pb, As, and Cr elevations were observed, with Cd, Ni, and Hg elevated only at higher concentrations.

### 2.2. Histological Analyses Results

**Figure 1:**
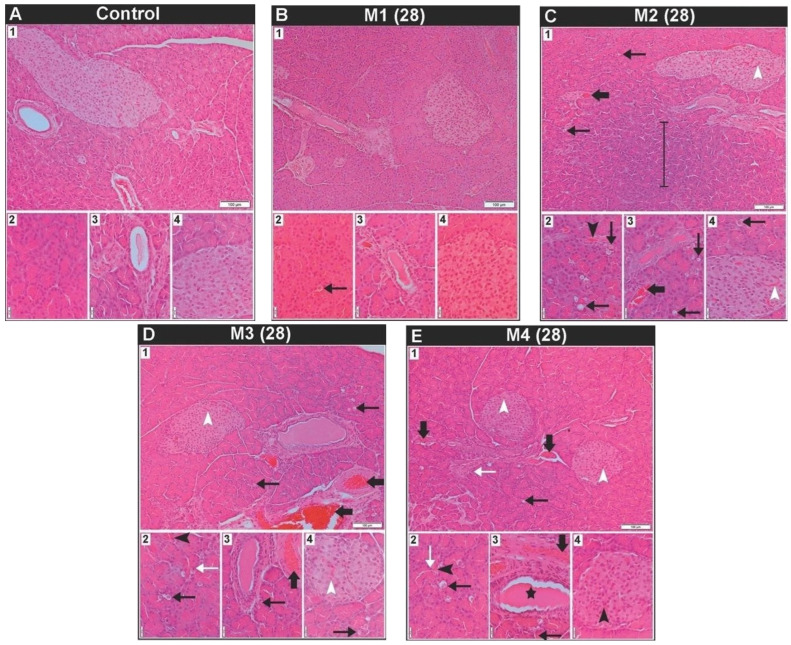
Representative photomicrographs of male Wistar rats’ pancreatic tissue section from the control group and groups treated with different doses of metals mixtures of Cd, As, Hg, Cr (VI), Pb, and Ni for 28 days; *n* = 5, per group. Doses based on: M1 group—calculated BMDL for effects on hormone levels; M2 group—median concentrations in human blood; M3 group—95th percentile concentrations in human blood; and M4 group—doses that were recalculated based on the reference values for individual metals found in the literature. (Thin black arrow—vacuolated acinar cell; thin white arrow—acinar cell with pyknotic nuclei; vertical line—exocrine pancreas area with increased basophilia and a reduction in the eosinophilic component of acinar cells; thick black arrow—vascular congestion in interlobular blood vessel; black arrowhead—capillary hyperaemia surrounding the acini; white arrowhead—capillary hyperaemia in the Langerhans islets; black star—dilated excretory duct.) Sections stained with haematoxylin/eosin were viewed with a low-power (10×) objective lens (**A_1_**–**E_1_**) (bar: 100 μm) and a high-power (40×) objective lens (**A_2_**_–_**A_4_**–**E_2_**_–_**E_4_**) (bar: 20 μm).

The histological characteristics of the pancreas in male rats following 28 days of exposure to toxic metal(oid) mixtures are presented in Figure 1. The pancreas of control animals exhibited a normal architecture predominantly comprising exocrine tubuloacinar tissue and endocrine Langerhans islets, which were distributed throughout the lobules. The acinar serous cells displayed two distinct regions: a basal region characterized by basophilic cytoplasm and a nucleus, and an apical region with acidophilic cytoplasm rich in zymogen granules. Langerhans islets were characterized by pale-staining endocrine cells organized in cords associated with capillary networks. The connective tissue septa contained interlobular blood vessels and excretory ducts (Figure 1(A1–A4)). On tissue sections from all treated groups there was evident cytoplasmic vacuolization of acinar cells (Figure 1(B2,C1–C4,D1–D4,E1–E3)). Acinar cell cytoplasmic vacuolization was least pronounced in the M1 group (Figure 1(B2)), but in the M3 and M4 group additionally, there were acinar cells with pyknotic nuclei (Figure 1(D2,E1,E2)). In the M2 group in certain regions there were areas of the exocrine pancreas with increased basophilia and a reduction in the eosinophilic component of acinar cells (Figure 1(C1)). Also, in the M2 group, and in the M3 and M4 groups there was evidence of vascular congestion in interlobular blood vessels (Figure 1(C1,C3,D1,D3,E1,E3)), as well as mild hyperaemia in capillaries surrounding the acini and in the capillaries of Langerhans islets (Figure 1(C1,C2,C4,D1,D2,D4,E1,E2,E4)). Additionally, in the M4 group, dilatation of the excretory ducts was observed (Figure 1(E3)).

**Figure 2:**
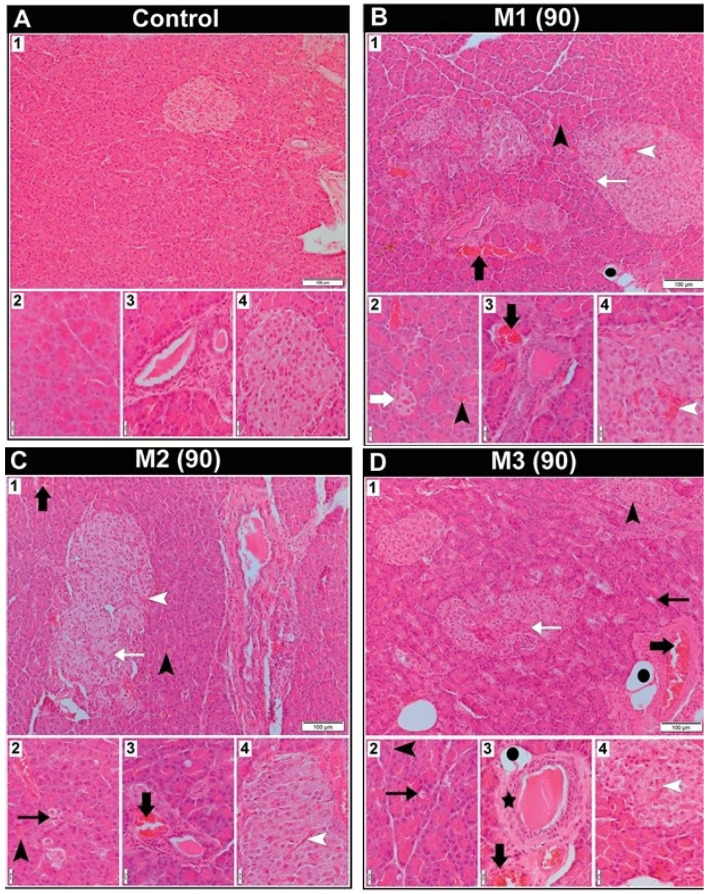
Representative photomicrographs of male Wistar rats’ pancreatic tissue section from the control group and groups treated with different doses of metal mixtures of Cd, As, Hg, Cr (VI), Pb, and Ni for 90 days; *n* = 5, per group. Doses based on: M1 group—calculated BMDL for effects on hormone levels; M2 group—median concentrations in human blood; and M3 group—95th percentile concentrations in human blood. (Thin black arrow—vacuolated acinar cell; thin white arrow—connective tissue cord in the Langerhans islets; thick black arrow—vascular congestion in interlobular blood vessels; thick white arrow—small Langerhans islet; black arrowhead—capillary hyperaemia surrounding the acini; white arrowhead—capillary hyperaemia in the Langerhans islets; black dot—adipocyte; black star—excretory duct surrounded by a thicker connective tissue layer.) Sections stained with haematoxylin/eosin were viewed with a low-power (10×) objective lens (**A_1_**–**D_1_**) (bar: 100 μm) and a high-power (40×) objective lens (**A_2_**_–_**A_4_**–**D_2_**_–_**D_4_**) (bar: 20 μm).

The histological characteristics of the pancreas in male rats following 90 days of exposure to toxic metal(oid) mixtures are presented in Figure 2. The rats from the control group had a pancreas with typical histoarchitecture (Figure 2(A1–A4)). All treated groups exhibited vascular congestion in interlobular blood vessels and hyperaemia of Langerhans islet capillaries and capillaries surrounding the acini (Figure 2(B1–B4,D1–D4)). Additionally, in all treated groups, most Langerhans islets were well-defined and oval, but some islets with an irregular shape were imbued with connective tissue cords (Figure 2(B1–D1)). It was noticeable that the Langerhans islets were smaller and diffusely dispersed in all treated groups compared to the control group, but most markedly in the M1 group (Figure 2(B2)). In the M1 and M3 groups, rare adipocytes were observed near the connective tissue septa and within the parenchyma (Figure 2(B1,D1,D3)). Acinar cytoplasmic vacuolization was identified in the M2 and M3 groups (Figure 2(C2,D1,D2)). In the M3 group, it was also noted that some of the excretory ducts were surrounded by a thicker layer of connective tissue (Figure 2(D3)).

**Figure 3:**
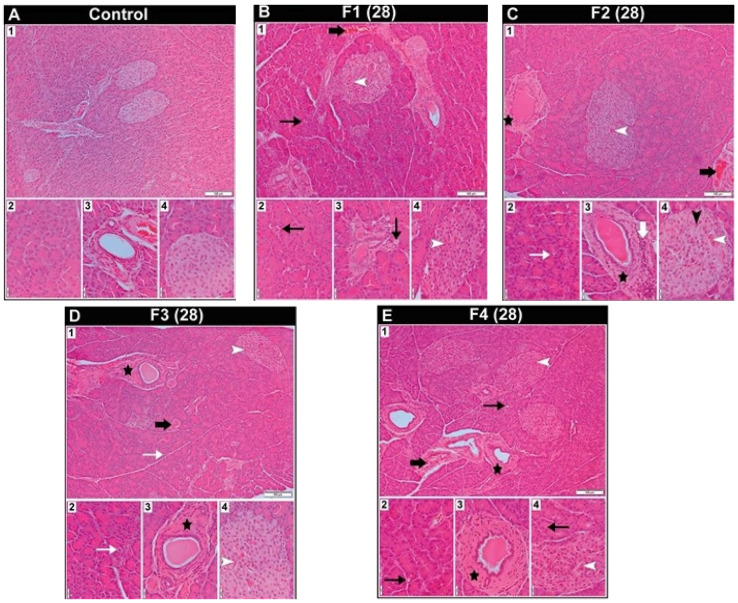
Representative photomicrographs of female Wistar rats’ pancreatic tissue section from the control group and groups treated with different doses of metal mixtures of Cd, As, Hg, Cr (VI), Pb, and Ni for 28 days; *n* = 5, per group. Doses based on: F1 group—calculated BMDL for effects on hormone levels; F2 group—median concentrations in human blood; F3 group—95th percentile concentrations in human blood; and F4 group—doses that were recalculated based on the reference values for individual metals found in the literature. (Thin black arrow—vacuolated acinar cell; thin white arrow—acinar cell with pyknotic nuclei; thick black arrow—vascular congestion in interlobular blood vessels; black arrowhead—endothelial cell with a basophilic nucleus in the capillary of the Langerhans islets; white arrowhead—capillary hyperaemia in the Langerhans islets; thick white arrow—increased cellularity of the connective tissue sheath of the excretory duct; black star—excretory duct surrounded by a thick connective tissue layer.) Sections stained with haematoxylin/eosin were viewed with a low-power (10×) objective lens (**A_1_**–**E_1_**) (bar: 100 μm) and a high-power (40×) objective lens (**A_2_**–**A_4_**–**E_2_**–**E_4_**) (bar: 20 μm).

The histological characteristics of the pancreas in female rats following 28 days of exposure to toxic metal(oid) mixtures are presented in Figure 3. The pancreas in the control group showed regular tissue organization (Figure 3(A1–A4)). Vascular congestion of interlobular blood vessels and hyperaemia of Langerhans islet capillaries were common characteristics in all treated groups (Figure 3(B1,B4–E1,E4). In the F1 and F4 groups, vacuolated acinar cells were visible (Figure 3(B1–B3,E1,E2,E4)), while in the F2 group, acinar cells with pyknotic nuclei were present (Figure 3(C2)). It was observed that the endothelial cells of the capillaries in the islets of Langerhans in the F2 group had prominent basophilic nuclei protruding into the capillary lumen (Figure 3(C4)). In the F2, F3, and F4 groups, a significant amount of connective tissue was observed around some of the excretory ducts (Figure 3(C1,C3–E1,E3)), but in the F2 group, this connective tissue exhibited pronounced cellularity (Figure 3(C3)).

**Figure 4:**
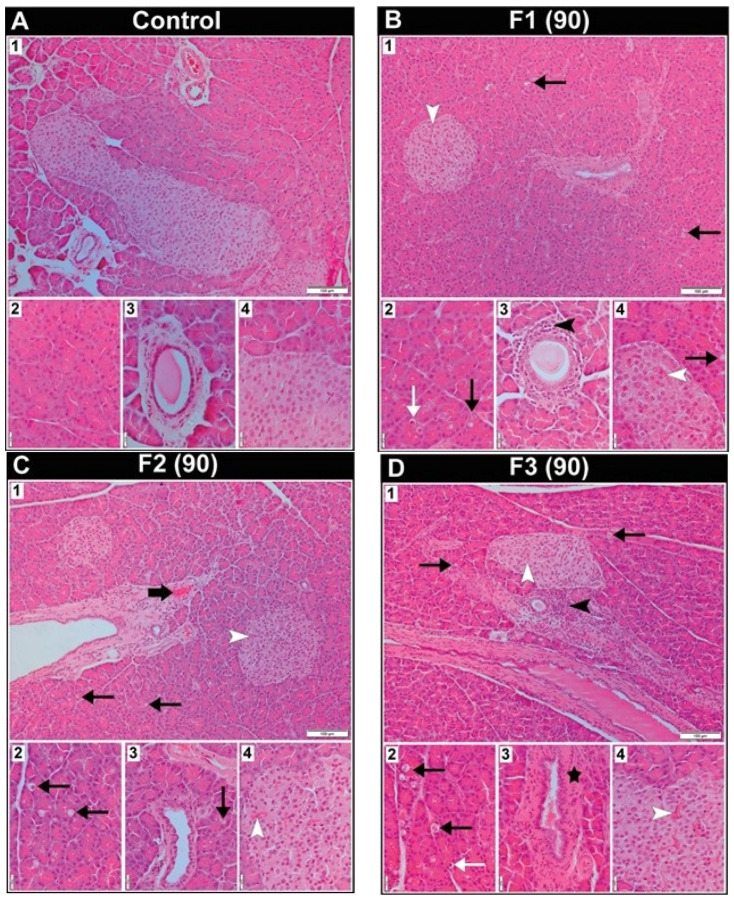
Representative photomicrographs of female Wistar rats’ pancreatic tissue section from the control group and groups treated with different doses of metal mixtures of Cd, As, Hg, Cr (VI), Pb, and Ni for 90 days; *n* = 5, per group. Doses based on: F1 group—calculated BMDL for effects on hormone levels; F2 group—median concentrations in human blood; and F3 group—95th percentile concentrations in human blood. (Thin black arrow—vacuolated acinar cell; thin white arrow—acinar cell with pyknotic nuclei; thick black arrow—vascular congestion in interlobular blood vessels; black arrowhead—mononuclear cell infiltrate; white arrowhead—capillary hyperaemia in the Langerhans islets; black star—excretory duct surrounded by a thick connective tissue layer.) Sections stained with haematoxylin/eosin were viewed with a low-power (10×) objective lens (**A_1_**–**D_1_**) (bar: 100 μm) and a high-power (40×) objective lens (**A_2_**–**A_4_**–**D_2_**–**D_4_**) (bar: 20 μm).

The histological characteristics of the pancreas in female rats following 90 days of exposure to toxic metal(oid) mixtures are presented in Figure 4. The pancreas of the rats from the control group displayed the characteristic structure typical for this gland (Figure 4(A1–A4)). Vacuolated cells of the exocrine acini were present in all treated groups (Figure 4(B1–B3,C1–C3,D1,D2)), but they were most pronounced in the F3 group (Figure 4(D1,D2)). Exocrine acinar cells with pyknotic nuclei were noteworthy in the F1 and F3 groups (Figure 4(B2,D2)). The hyperaemia of Langerhans islet capillaries was evident in all treated groups (Figure 4(B1,B4–D1,D4)), but the vascular congestion of the interlobular blood vessels was present only in the F2 group (Figure 4(C1)). Certain excretory ducts were surrounded by a mononuclear cell infiltrate in the F1 and F3 groups (Figure 4(B3,D1)), while an abundant connective tissue sheath around some excretory ducts was observed in the F3 group (Figure 4(D3)).

Overall, male rats exhibited more pronounced vascular congestion and connective tissue alterations after 90 days, with Langerhans islets becoming smaller, diffusely distributed, and partially replaced by connective tissue. Females showed less consistent connective tissue changes and more prominent acinar vacuolization. Comparing 28 and 90 days, histological damage intensified over time, with more severe vascular, acinar, and connective tissue changes in both sexes, but males demonstrated greater structural remodelling of Langerhans islets, while females exhibited more acinar cell alterations.

### 2.3. Glucose Levels

**Figure 5:**
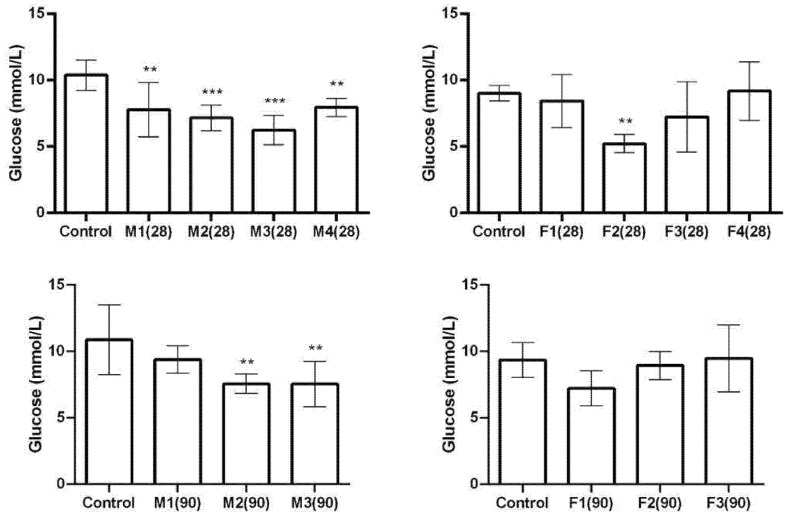
Serum glucose levels of male and female rats after 28/90 days of exposure to a mixture of toxic metals (As, Pb, Hg, Cd, Cr (VI), and Ni) at four different doses calculated based on a previous human biomonitoring study (doses based on: M1/F1—calculated lower confidence limit of the Benchmark dose for effects on hormone levels; M2/F2—median concentration in human blood; M3/F3—95th percentile concentration in human blood; and M4/F4—reference doses). ANOVA + LSD test (graphs show mean ± SD). ** *p* < 0.01; *** *p* < 0.001 (compared to control).

After 28 days of exposure, glucose levels decreased in all the treated groups of male animals (Figure 5). In female animals, glucose levels were reduced only in the F2 group compared to the control. After 90 days of exposure, glucose levels were lower in male animals in groups M2 and M3 but not in M1. On the other hand, no significant changes in glucose levels were observed in any of the female groups.

### 2.4. MDH-1 Activity

**Figure 6:**
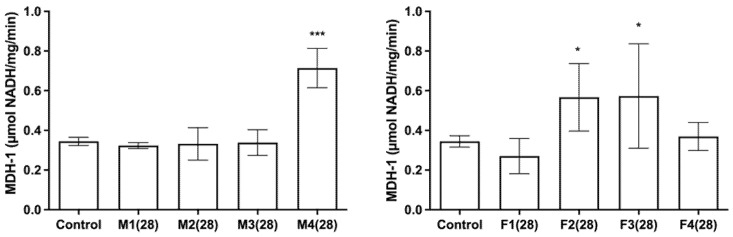

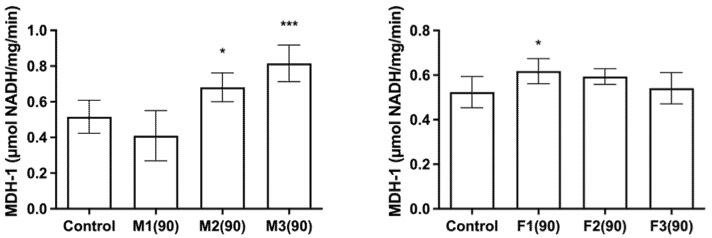
Malate dehydrogenase-1 (MDH-1) levels in the pancreas of male and female rats after 28/90 days of exposure to a mixture of toxic metals (As, Pb, Hg, Cd, Cr (VI), and Ni) at four different doses calculated based on a previous human biomonitoring study (doses based on: M1/F1—calculated lower confidence limit of the Benchmark dose for effects on hormone levels; M2/F2—median concentration in human blood; M3/F3—95th percentile concentration in human blood; and M4/F4—reference doses). ANOVA + LSD test (graphs show mean ± SD). * *p* < 0.05; *** *p* < 0.001 (compared to control).

Measured MDH-1 activity in male and female rats after a 28- and 90-day exposure to toxic metal mixtures is shown in Figure 6. In male rats after 28 days of exposure, elevated MDH-1 activity was observed only in the group receiving the highest dose levels of toxic metals (M4). In female rats, on the other hand, elevated levels were noted in F2 and F3. After 90 days of exposure, in male rats, higher MDH-1 activity was seen in M2 and M3. In female rats after 90 days of exposure, an increase was observed only in F1.

### 2.5. Amylase Activity

**Figure 7:**
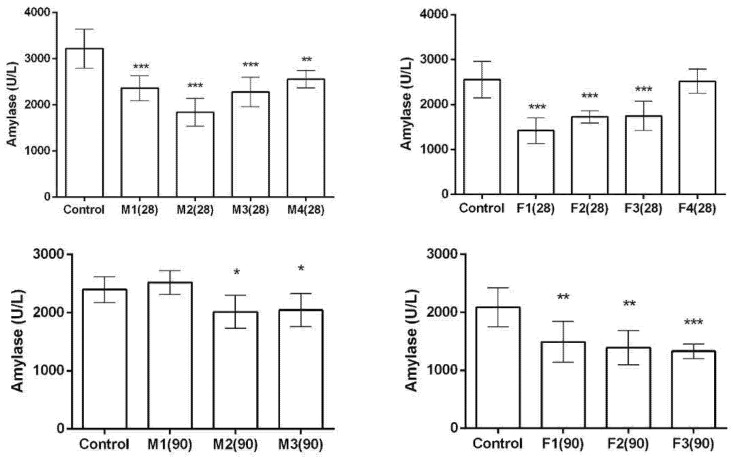
Amylase levels in the serum of male and female rats after 28/90 days of exposure to a mixture of toxic metals (As, Pb, Hg, Cd, Cr (VI), and Ni) at four different doses calculated based on a previous human biomonitoring study (doses based on: M1/F1—calculated lower confidence limit of the Benchmark dose for effects on hormone levels; M2/F2—median concentration in human blood; M3/F3—95th percentile concentration in human blood; and M4/F4—reference doses). ANOVA + LSD test (graphs show mean ± SD). * *p* < 0.05; ** *p* < 0.01; *** *p* < 0.001 (compared to control).

Measured amylase activity in male and female rats after a 28- and 90-day exposure to toxic metal mixtures is shown in Figure 7. After 28 days of exposure to the toxic metal mixture, male animals exhibited lower amylase levels across all treated groups. In female animals, amylase levels were also lower in all treated groups except for group F4. Following 90 days of exposure, amylase levels in male animals were reduced only in groups M2 and M3. In female animals, amylase levels were lower compared to the control in all treated groups, with the lowest levels observed in group F3.

### 2.6. Oxidative Stress/Antioxidant Protection Parameters

Oxidative stress and antioxidant protection parameters in the pancreas of male and female rats after 28/90 days of exposure to a mixture of toxic metals are presented in Table 2.

After 28 days of exposure to toxic metal mixtures, male animals in the M2 and M4 groups exhibited significantly higher IMA levels, while this change was more prominent in M4. MDA levels were significantly reduced only in the M1 group. Additionally, SH levels were significantly higher in the M1 group. Although changes in GSH and SOD were not statistically significant, there was a notable trend of lower values in the treated groups compared to the control. In female animals, IMA levels were significantly higher in the M4 group compared to the control. MDA levels were significantly elevated only in the F2 group. Additionally, SH groups and GSH levels were both lower in the F2 group. However, there were no significant changes observed in SOD levels. In male rats, after 90 days of exposure, no statistically significant changes were observed in any of the measured parameters among the treated groups compared to the control. The applied metal mixtures showed trends of increased MDA concentrations in M2 and M3, and slightly increased SOD concentrations in M2 and M3. In female rats following a 90-day exposure, a trend of increased MDA levels was noted across the treated groups. However, these changes did not reach statistical significance. A statistically significant increase in IMA and SH group levels was observed in the F1 group. Furthermore, GSH was significantly increased in the F2 group, while SOD activity was elevated in F2 and F3.

### 2.7. Bioelements

The levels of bioelements measured in rat pancreatic tissue are presented in Table 3. After a 28-day exposure in male rats, Fe levels decreased in groups M2, M3, and M4, while Cu levels decreased in all groups, with the least decrease observed in M4 and a similar decrease across M1, M2, and M3. Zn levels decreased uniformly across all groups. Mn levels were notably reduced in groups M1, M2, and M3, with the most significant decrease observed in M3; however, this reduction did not occur in group M4. Similarly, Ca levels decreased in groups M1, M2, and M3 but remained unchanged in group M4. On the other hand, Mg levels were elevated only in group M4. In female rats subjected to a 28-day exposure, only Ca and Mn levels decreased in the F1 group, while other bioelements showed no significant changes. In male rats, after a 90-day exposure, Fe levels decreased solely in group M2, while Cu levels decreased in both M1 and M2. Zn levels decreased across all groups, with a lesser decrease in M2 and a similar decrease in M1 and M3. Ca levels decreased in groups M2 and M3, and Mg levels exhibited a decrease in all exposure groups (M1, M2, and M3) compared to the control. Mn levels, however, showed no significant changes across any of the exposure groups in comparison with the control. After a 90-day exposure in female rats, Mg levels decreased in all exposure groups compared to the control, with a more pronounced decrease in F2 and F3. Similarly, Ca levels decreased uniformly across all groups, while Zn levels decreased only in the highest concentration group (F3). However, no significant changes were observed in Mn, Fe, or Cu levels.

### 2.8. Correlation Matrix

**Figure 8:**
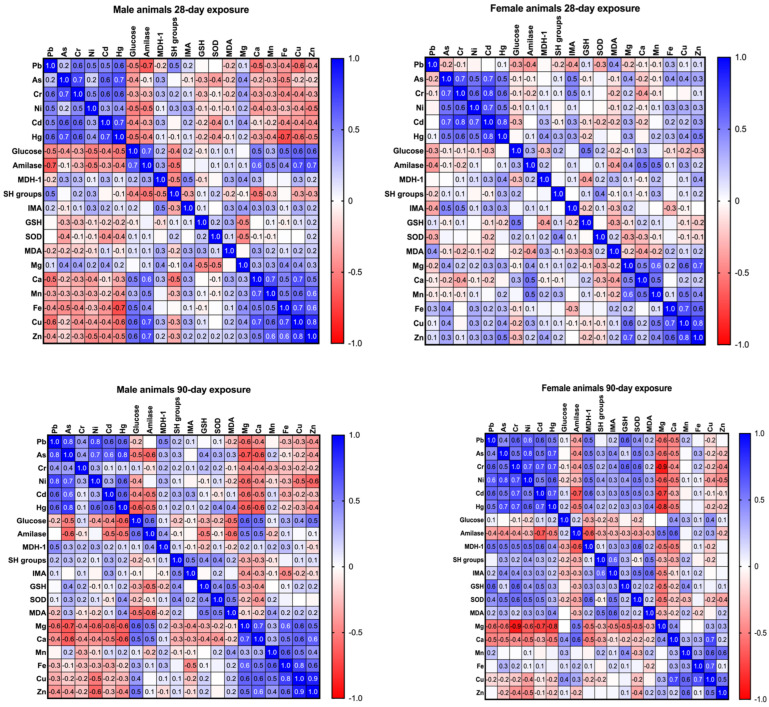
Correlation matrix showing oxidative stress markers, toxic metal levels, bioelements, serum glucose, and amylase activity after 28 and 90 days of exposure in male and female animals (positive correlations: blue; negative correlations: red; weak correlations: white or light shades; strong correlations: darker colours).

In Figure 8, a correlation matrix is shown. Toxic metals, glucose levels, amylase and MDH-1 activity, and bioelements are compared after 28 days and 90 days of exposure in male and female animals. The colours and numbers in the figure represent the strength and direction of correlations between the different variables. The coefficient ranges from −1 to 1 and indicates how strongly two variables are related. Positive correlations are shown in blue, with darker shades indicating stronger relationships where both variables increase together. Negative correlations are shown in red, with darker shades indicating stronger inverse relationships. Weak correlations (r near 0) are shown in white or light shades, indicating minimal or no relationship.

## 3. Discussion

### 3.1. Study Rationale and Key Findings

The recently introduced Real-Life Risk Simulation (RLRS) concept provides a valuable method for assessing the impact of low-dose chemical mixtures, reflecting realistic population exposure scenarios [30,31]. Based on this concept, our research group previously conducted biomonitoring studies that identified associations between toxic metals and metabolic parameters, including positive correlations of As, Pb and Hg with serum insulin and a negative correlation for Ni, suggesting their potential role as metabolic disruptors [27,29]. These findings informed the design of subsequent animal studies using human-relevant exposure conditions. In these studies, metal mixture induced sex- and exposure duration-dependent endocrine and reproductive disturbances, including alterations in thyroid hormones, reproductive hormones, and redox status [32,33]. Collectively, these findings indicate that toxic metal mixtures can disrupt multiple physiological systems and provide a rationale for investigating pancreatic structure and function under real-life exposure conditions. In line with this, some researchers have investigated the effects of toxic metal mixtures on pancreatic function in both in vitro and in vivo settings. In INS-1 β-cells, mixtures containing As and Mn impaired glucose-stimulated insulin secretion and mitochondrial function, indicating disrupted β-cell metabolism and mitochondrial targeting [34,35]. Several in vivo studies have further demonstrated that mixed heavy metal exposure can induce metabolic disturbances, pancreatic damage, and systemic toxicity in animal models. To investigate the combined effects of As, Cd, Pb, Cr, and Hg, Zhu et al. (2014) [36] reported increased glucose levels in female rats after a 90-day exposure to a mixture of As, Cd, Pb, Cr, and Hg. Similarly, prenatal exposure to Pb-, Cd-, Mn-, and As-containing mixtures induced gestational diabetes mellitus-like disturbances, including hyperglycaemia, insulin resistance, and dyslipidaemia [37]. These findings are further supported by studies showing altered glucose homeostasis following prolonged exposure to more complex metal mixtures. For example, a 90-day exposure to mixtures containing Zn, Cu, Mn, Cr, Ni, Cd, Pb, and Hg decreased glucose levels at higher doses [38]. In addition to metabolic alterations, several studies have reported direct structural pancreatic damage following combined metal exposure. Riaz et al. (2020) [39] demonstrated that combined exposure to Pb, Mn, Cd, and As induced degeneration and necrosis of pancreatic islets of Langerhans, accompanied by reduced pancreatic Bcl2 expression. Beyond pancreatic alterations, exposure to a Pb–Hg mixture also promoted hepatic insulin resistance, oxidative stress, inflammation, and dyslipidaemia in rats [40]. Moreover, combined metal exposure may aggravate pre-existing metabolic disorders. In streptozotocin-induced diabetic rats, co-exposure to Cd and As disrupted glucose and amylase levels and induced kidney and liver damage, indicating aggravated metabolic and organ toxicity [41]. However, most of these studies used substantially higher doses than those applied in the present study. In this context, the present study demonstrates that real-life metal mixture exposure induces sex- and time-dependent pancreatic toxicity characterized by metal accumulation, histopathological damage, metabolic disruption, oxidative stress, and mineral imbalance. Males showed more pronounced early disturbances with partial adaptation over time, whereas females exhibited more sustained oxidative stress with compensatory antioxidant responses. These effects were accompanied by alterations in glucose metabolism, amylase activity, and MDH-1 function, as well as dose-dependent metal accumulation and disrupted bioelement homeostasis. These findings are further discussed in the following sections.

### 3.2. Pancreatic Accumulation of Toxic Metals

Studies from the literature have suggested the property of toxic metals to accumulate in pancreatic tissue after the exposure. Our research group previously reported significant pancreatic Cd accumulation after both single and repeated Cd exposure in rats [42]. Similarly, subcutaneous Cd exposure increased pancreatic Cd levels in a dose-dependent manner and induced glucose intolerance at higher doses [43]. Pancreatic accumulation has also been reported following Pb exposure through drinking water [44], dietary Cr supplementation [45], and As exposure, which was additionally associated with oxidative stress, hyperglycaemia, and impaired glucose tolerance [35]. In the current study, pancreatic accumulation of toxic metals was both sex- and exposure-duration-dependent. After 28 days of exposure, males generally exhibited greater and more dose-dependent accumulation of Pb, Cd, Hg, and As compared to females, which showed more selective increases mainly at higher exposure levels. Following 90 days of exposure, accumulation patterns became more pronounced in both sexes, although females demonstrated broader increases in Pb, As, and Cr across exposure groups, while Cd, Ni, and Hg elevations remained primarily associated with higher doses. Overall, these findings indicate differential susceptibility and accumulation dynamics between male and female rats during prolonged exposure to the metal mixture.

### 3.3. Histopathological Alterations, and Metabolic and Functional Pancreatic Impairment

On the basis of histopathological results, it can be concluded that both male and female rats exhibited dose-dependent changes, with acinar cell vacuolization, vascular congestion, and alterations in Langerhans islet structure becoming more pronounced at higher doses and longer exposure durations. These changes were evident as early as 28 days and persisted or intensified after 90 days. In general, male rats showed greater vascular congestion and connective tissue changes after 90 days, with Langerhans islets becoming smaller, more dispersed, and partly replaced by connective tissue. In contrast, females exhibited less consistent connective tissue alterations but more prominent acinar vacuolization, along with occasional mononuclear cell infiltration. To further assess whether metal exposure affected pancreatic function and the levels of glucose, amylase, and MDH-1, valuable biomarkers for pancreatic health were measured and analysed. Glucose serves as the primary energy source in the human body, while its levels indicate how well the pancreas regulates blood sugar through insulin and glucagon secretion [46,47]. Amylase, a pancreatic enzyme, has a role in carbohydrate digestion. Elevated amylase is associated with pancreatic, salivary, and intestinal disorders [48]. MDH-1 is a key enzyme in the malate–aspartate shuttle. This process regenerates cytosolic NAD+, which is necessary for continuous glycolysis and ATP production. Generated ATP is a primary coupling factor for insulin secretion in pancreatic beta cells [49,50]. After 28 days, glucose decreased in all treated male groups, while in females it decreased only in F2. After 90 days, glucose was reduced in males in higher dose groups, with no changes in females. MDH-1 activity increased in the highest dose group in males and in F2–F3 in females after 28 days, while after 90 days it increased in M2–M3 in males and only in the lowest dose group in females. Amylase decreased in all treated male groups at 28 days and in higher dose groups at 90 days, whereas females showed reductions in most groups at both time points, with the lowest levels in F3.

### 3.4. Oxidative Stress, Antioxidant Response, and Mineral Homeostasis Disruption

We further measured oxidative stress parameters as key mediators of cellular damage to assess their role in metal-induced toxicity. Antioxidant markers were also analysed to better understand the balance between oxidative stress and antioxidant defence mechanisms. Oxidative stress is central to disease development, while disruption of redox balance is a key mechanism of heavy metal toxicity [51]. Additionally, it worsens diabetes by impairing β-cell function and insulin sensitivity, disrupting glucose regulation, and contributing to vascular damage and complications such as retinopathy, nephropathy, and cardiovascular disease [52]. It has also been demonstrated that toxic metals can interfere metabolically with essential nutritional metals. For instance, Pb disrupts Ca in the nervous system and replaces Zn in heme enzymes, while Cd affects Ca levels in the skeletal system and substitutes Zn in metallothioneins [53]. Therefore, bioelement levels in pancreatic tissue were measured to comprehensively assess these interactions. The results confirm that toxic metal mixture exposure induces oxidative stress and disrupts mineral homeostasis. Males showed stronger oxidative and metabolic alterations at 28 days, followed by partial adaptation at 90 days, whereas females maintained oxidative stress with compensatory antioxidant responses. At 28 days, males exhibited increased IMA, reduced MDA (linked to higher SH), decreased bioelements, and lower amylase and glucose, indicating metabolic disruption. Females showed increased MDA, reduced SH/GSH, and lower glucose, with limited Ca and Mg changes. After 90 days, males showed no significant oxidative changes, while females exhibited elevated IMA and antioxidant markers but persistent reductions in Ca and Mg, with decreased Zn in the highest exposure group. These results are slightly different than the results of our previous study, which revealed that a 90-day exposure to a real-life mixture of toxic metals depleted GSH levels in males, elevated IMA levels in male livers, and increased MDA levels in kidneys of both male and female animals, with more pronounced effects in males, highlighting sex-specific hepato-renal toxic mechanisms [54].

### 3.5. Metal Co-Accumulation and Disruption of Pancreatic Homeostasis

After evaluating the investigated parameters individually, correlation analysis was performed to identify patterns of co-accumulation and potential disruptions in homeostasis following prolonged toxic metal exposure. Across both exposure durations (28 and 90 days), consistent correlations were observed among toxic metals as well as among bioelements. Correlations between toxic metals in pancreatic tissue suggest co-accumulation, while correlations among bioelements indicate maintained mineral balance despite exposure. In males, after 28 days, the negative correlation between pancreatic Pb and amylase suggests impaired pancreatic enzyme function. After 90 days, associations between glucose, amylase, and Mg indicate progressive effects on pancreatic function and glucose metabolism. Positive correlations between amylase, As, and MDA further suggest a link between As accumulation and oxidative pancreatic damage, while correlations between As and Mg/Ca indicate disrupted mineral homeostasis. In females, correlations after 28 days were generally weaker than in males, apart from those among toxic metals. However, after 90 days, correlations became more pronounced, particularly the negative correlations between toxic metals and Mg, especially Cr and Mg, suggesting disruption of essential mineral balance. Positive correlations between Cr and antioxidant markers (GSH and SOD) may reflect an adaptive oxidative stress response.

### 3.6. Sex- and Exposure-Duration-Dependent Susceptibility to Metal Mixture Toxicity

The results of the current study indicate sex- and exposure-duration-dependent differences in response to metal mixture exposure. Similar sex-specific differences in pancreatic function have been reported previously. In a rat study investigating oxidative stress-related insulin resistance, males showed a more pronounced insulin resistance profile, whereas females exhibited higher SOD-2 and uncoupling protein 2 levels, lower oxidative damage, and larger islets, supporting sex dimorphism in pancreatic oxidative status [55]. Another study showed that male mice develop more severe damage during acute pancreatic inflammation, including distorted endoplasmic reticulum structure and greater inflammation compared to females [56]. Furthermore, a study utilizing mice with pancreas-specific Atg5 disruption to examine chronic pancreatitis revealed a higher percentage of disease development in males, with more pronounced pancreatic damage and inflammation than in females [57]. In our study, sex-dependent differences were observed in both histopathological and functional pancreatic outcomes. Males showed more pronounced vascular changes at 28 days, followed by an apparent shift toward adaptation after 90 days, whereas females exhibited more persistent structural and oxidative alterations with signs of compensatory antioxidant responses. These patterns were also reflected at the functional level, with males demonstrating greater reductions in glucose and amylase compared to females. These findings are consistent with our previous study, which demonstrated more pronounced redox disturbances in the liver and kidney tissue of males following exposure to a real-life metal mixture [54]. These results indicate that males and females may differ in susceptibility and response mechanisms to toxic metal(oid) exposure, with males adapting more acutely over time and females showing persistent stress-related effects.

### 3.7. Limitations and Further Directions

Several limitations should be acknowledged in the present study. First, the investigated mixture included only six metals, whereas real-life exposure scenarios involve a much broader range of environmental contaminants, such as pesticides, phthalates, and mycotoxins. Second, the study was limited to two exposure time points (28 and 90 days), which precludes evaluation of long-term or chronic effects. Furthermore, the islets of Langerhans constitute only 1% of the pancreatic mass but receive 20% of its blood supply [58], which could result in higher metal accumulation in the endocrine pancreas compared to the exocrine tissue. However, metals were not specifically measured in the islets of Langerhans due to the challenge of isolating these structures from the surrounding exocrine tissue, making it impossible to assess potential differences in metal accumulation and their functional consequences, such as impaired insulin secretion or endocrine toxicity. Furthermore, insulin, which could have provided additional insights into pancreatic endocrine function and overall metabolic health, was not measured in this study due to the limited volume of available serum samples. Another limitation of this study is that histopathological evaluation was performed qualitatively without the use of a blinded semi-quantitative scoring system, which would have enabled a more objective and standardized comparison of tissue alterations.

Taking into account the limitations of the present study, our future research will focus on expanding the experimental design to include a broader spectrum of environmentally relevant contaminants beyond the six investigated metals, which will better reflect real-life exposure scenarios. In addition, longer exposure periods with multiple time points will be incorporated to enable assessment of chronic and cumulative effects. Moreover, additional biomarkers of endocrine function will be included to provide a more comprehensive evaluation of pancreatic metabolic status. BMD analyses of different real-life metal mixture compositions will also be incorporated to identify critical exposure thresholds and refine dose–response relationships across sexes. Finally, future research will explore potential protective strategies against environmental mixture toxicity, including antioxidant, metal-chelating, and cytoprotective compounds, with the aim of identifying effective interventions to mitigate adverse effects of combined real-life exposures.

## 4. Materials and Methods

The current study aimed to investigate the impact of toxic metal mixtures (As, Pb, Hg, Cd, Cr (VI), and Ni) on the following parameters in Wistar rats: (i) metal accumulation in pancreatic tissue; (ii) histopathological changes in pancreas tissue; (iii) serum levels of glucose, amylase, and pancreatic tissue malate dehydrogenase 1 (MDH-1) activity; (iv) oxidative stress markers and antioxidant defence mechanisms in pancreatic tissue; and (v) bioelement levels within the pancreatic tissue. The study focuses on sex-specific and duration-specific differences by evaluating these impacts after a 28- or 90-day exposure of male and female rats to doses that reflect real-life levels in the general population.

### 4.1. Experimental Animals

The animal research protocol was approved by the Ethics Committee at the Faculty of Pharmacy, University of Belgrade, with the approval number 323-07-11822/2018-05. The study complied with Serbia’s Animal Welfare Law and the European Union Directive EU2010/86. The study was conducted in accordance with the ARRIVE (Animal Research: Reporting of In Vivo Experiments) guidelines to ensure transparency and reproducibility [59]. In the study, male and female albino Wistar rats were used, aged between 6 and 7 weeks and weighing between 150 and 180 g. Animals were not genetically modified and had not undergone prior experimental procedures. The rat was selected as a model because its pancreas shares important anatomical and cellular features with the human pancreas, including ductal drainage into the duodenum and comparable populations of acinar, ductal, stellate, and endocrine cells, which together make it suitable for studying general pancreatic structure and function [60]. These rats were obtained from a licensed breeder affiliated with the Military Medical Academy in Belgrade, Serbia. Animals were housed in climate-controlled conditions within plexiglass cages (five animals per cage). The temperature was maintained between 20 and 24 °C, with air humidity kept at 40–60% and a consistent day/night cycle. All cages were maintained in a uniformly controlled environment throughout the experiment. The rats had unrestricted access to tap water and standard pellet-based food provided by the Veterinary Institute “Subotica”, Serbia. Environmental enrichment was provided in the form of bedding and nesting material. Prior to commencing the experimental procedures, the animals were given a seven-day period to acclimate. All animals were in good health and exhibited no signs of disease or abnormalities prior to the initiation of the experiment. Animals were monitored daily for signs of distress (e.g., reduced activity, weight loss, or abnormal behaviour), and no humane endpoints requiring early euthanasia were reached during the experiment.

### 4.2. Experimental Protocol

A total of 90 experimental animals were used (45 males and 45 females), divided into 18 groups (14 treated animals and 4 controls). Each experimental group consisted of 5 animals. The experimental unit was an individual animal. The animals were exposed to a toxic metal mixture for either 28 or 90 days. Two control groups, one for males and one for females, were given deionised water, while the remaining groups received aqueous solutions of toxic metal mixtures in four different doses, determined based on prior human biomonitoring research [26,27,28,29] (M—male animals; F—female animals). The lowest doses (M1/F1) were based on BMDL values associated with endocrine-related effects, selecting the lowest available value for each metal and prioritizing those with the narrowest confidence intervals to ensure greater precision; several of these endpoints were related to alterations in insulin levels. Specifically, for Cd, BMDLs for testosterone in men (0.273 μg/L) and insulin in women (0.0032 μg/L) were used; for Pb, free triiodothyronine (FT3) in men (0.397 μg/L) and women (0.629 μg/L); for As, insulin in men (1.22 μg/L) and women (0.88 μg/L); for Hg, insulin in men (0.0883 μg/L) and thyroid-stimulating hormone (TSH) in women (0.494 μg/L); for Cr, testosterone in men (0.0434 μg/L) and free thyroxine (FT4) in women (0.0121 μg/L); and for Ni, FT3 in men (1.0 μg/L) and insulin in women (0.0032 μg/L). The mid-dose levels (M2/F2) corresponded to median biomonitoring concentrations (µg/L): Cd, 1.476 in men and 1.962 in women; Pb, 3.495 and 22.528; As, 0.971 and 10.443; Hg, 5.219 and 3.444; Cr, 1.324 and 1.060; and Ni, 7.609 and 8.278. The highest doses (M3/F3) were based on the 95th percentile concentrations (µg/L): Cd, 4.28 in men and 6.45 in women; Pb, 99.55 and 139.41; As, 17.49 and 29.53; Hg, 48.89 and 30.76; Cr, 11.52 and 44.9; and Ni, 48.03 and 87.2. Each dose level had a corresponding group treated for 28 days and a group treated for 90 days. The exception was the M4/F4 group (which received metal doses based on the reference values for individual metals found in the literature), which was treated for 28 days only [61,62,63,64]. This was a choice guided by animal welfare standards and the 3R principle, given the elevated levels of toxic metals. Each group consisted of five rats. The number of animals was determined also in accordance with the 3R principle, while the statistical power of the study was additionally verified using the G*Power software 3.1 (Heinrich Heine University, Düsseldorf, Germany). Animals were randomly assigned to experimental groups using a simple randomization method, in which each animal was allocated according to a computer-generated random sequence. No predefined inclusion or exclusion criteria were applied. All animals allocated to the study were included in the analysis, and no data points were excluded. Throughout the experiment, animals were monitored for any signs of discomfort, and appropriate handling techniques were employed to reduce stress. To minimize potential confounding effects, animals were housed under identical environmental conditions, and treatments and sample collection were performed in a consistent manner across groups. Outcome assessment and data analysis were performed without blinding to group allocation. Based on the recommendations of the U.S. Environmental Protection Agency and utilizing the reverse dosimetry method, the concentrations measured in the human population were translated into equivalent human doses [65]. All calculated human doses were converted into corresponding animal doses using formulas based on body surface area, while accounting for species differences [66]. Experimental solutions and deionised water in control groups were administered orally via gavage daily for 28/90 days. Oral gavage was selected to ensure precise and controlled daily dosing. Animals were fasted for 12 h prior to euthanasia. Animals were euthanized under anaesthesia, administered via an intraperitoneal injection of ketamine (75 mg/kg body weight) and xylazine (10 mg/kg body weight). Blood samples were subsequently collected through cardiac puncture post-anaesthesia administration. Pancreatic tissue samples were collected for further analysis. The outcome measures were pancreatic histopathological alteration, biochemical and oxidative stress parameters, and metal tissue concentrations. This study was not pre-registered.

The groups of experimental animals and the corresponding doses are presented in Table 4.

### 4.3. Toxic Metal and Bioelement Measurements

Rat pancreatic tissue samples were weighed and placed into Teflon vessels. Subsequently, 7 mL of concentrated nitric acid (cHNO_3_) and 1 mL of hydrogen peroxide (H_2_O_2_) were introduced. Microwave-assisted digestion was performed using a Milestone STARTD, SK-10T system (Milestone Srl, Sorisole, Italy). The mineralization process consisted of three phases: (I) gradual heating to reach 180 °C within 15 min, (II) sustaining this temperature for an additional 15 min, and (III) cooling and ventilating the apparatus for 30 min. After digestion, the mineralized samples were transferred to 25 mL volumetric flasks and diluted with deionized water to achieve the target volume. A blank sample was prepared by combining the oxidizing reagents (cHNO_3_ and H_2_O_2_) in a 7:1 volume ratio. All toxic metals were measured in digested samples using an ICP-MS NexION 300S (PerkinElmer, Waltham, Massachusetts, United States), a mass spectrometer with inductively coupled plasma (ICP) for multi-element analysis. The system operates in three modes—standard, collision, and reaction. It covers a mass range from 1 to 285 atomic mass units (amu) with a mass resolution of 0.3 amu, and it can detect elements from ng/L to mg/L concentrations. The instrument uses a 27 MHz RF generator with plasma power adjustable up to 1600 W. Calibration standards included PerkinElmer Instrument Calibration Standard 2 (100 µg/mL) for elements and a PerkinElmer Mercury (Hg) Pure Plus Standard at 1 µg/mL. The limits of detection (LOD) and quantification (LOQ) were 2 ppt and 20 ppt, respectively, for all the measured metals.

### 4.4. Histological Analyses

Pancreatic tissue samples were preserved for 24 h at room temperature in 10% neutral buffered formalin (pH 6.8). Following this preservation step, the tissues were processed and embedded in paraffin blocks using standard laboratory methods. Sections with a thickness of 5 μm were cut with a rotary microtome (Reichert, Wien, Austria). These sections were subsequently stained with haematoxylin/eosin (H/E) (Merck Millipore, Darmstadt, Germany) and mounted using DPX (phthalate-free) mounting medium (Fisher Scientific, Loughborough, UK). The stained sections were analysed for any changes in the pancreatic tissue structure under a microscope equipped with a digital camera and specialized software (Olympus CX31 with UC50 Soft Imaging Solutions camera and SensEntry 1.13 software, Münster, Germany).

### 4.5. Glucose Level and Amylase Activity Determination

Glucose levels and amylase activity were measured in serum. To obtain serum, whole blood samples were allowed to clot at room temperature for 30 min, followed by centrifugation at 3000× *g* for 30 min at room temperature (20–25 °C). Determination of these parameters was performed using commercial reagents on a Beckman Coulter AU 480 automatic analyser (Beckman Coulter, Brea, CA, USA). Calibration was carried out with commercial calibrators in accordance with NIST (National Institute of Standards and Technology) standard reference materials and the Beckman Coulter Master Calibrator.

### 4.6. MDH-1 Determination

For the analysis of MDH-1 pancreas concentration, tissue homogenates were prepared. To eliminate the remaining blood, the tissue was washed in cold PBS (0.01 M, pH = 7.4) and homogenised (also in PBS (tissue weight (g): PBS volume (mL) =1:9)) using a T10 basic UltraTurrax homogeniser (IKA, Königswinter, Germany). The homogenates were centrifuged at 5000× *g* for 5 min at 2–8 °C, and the supernatant was obtained to perform the test. MDH-1 determination was performed by a commercial rat ELISA kit (Abcam, Cambridge, UK), following the instructions of the manufacturer. The measurement was performed on a SPECTROstar Nano UV/VIS spectrophotometer (BMG LABTECH, Ortenberg, Germany).

### 4.7. Parameters of Oxidative Stress and Antioxidant Defence

To determine oxidative stress parameters and antioxidant defence, tissue samples were partially thawed on ice (0–4 °C). Samples weighing 0.2–0.4g were homogenized in a cold phosphate buffer (0.1 mol/L, pH 7.4, with 1.15% KCl) at a ratio of 1:9 (*v*/*v*) using a T10 basic ULTRA-TURRAX homogenizer (IKA, Staufen, Germany). The homogenates were centrifuged at 800× *g* for 10 min at +4 °C, followed by a second centrifugation at 9500× *g* for 20 min at +4 °C. The post-mitochondrial supernatant was then collected, separated into Eppendorf tubes, and used for the analysis of oxidative stress biomarkers. The following oxidative stress and antioxidant defence parameters were measured in pancreatic tissue homogenates: malondialdehyde (MDA), ischemia-modified albumin (IMA), sulfhydryl groups (SH groups), glutathione (GSH), and superoxide dismutase (SOD). These parameters were analysed spectrophotometrically using a SPECTROstar Nano UV/VIS spectrophotometer (BMG LABTECH, Ortenberg, Germany). Detailed methods for their measurement are provided in Table 5.

### 4.8. Statistical Analysis

Statistical data analysis and visualization were carried out with the GraphPad Prism 8 software (GraphPad Software, Inc., San Diego, CA, USA). Initially, the Shapiro–Wilk test was used to check the normality of the data distribution. For data showing a normal distribution, one-way ANOVA was employed, and Fisher’s Least Significant Difference (LSD) test was used for subsequent analysis of significant differences. For data that did not follow a normal distribution, the Kruskal–Wallis test was applied, followed by Dunn’s post hoc test. For data that followed a normal distribution, the results were presented as average values with standard deviations (SDs) to provide an accurate representation of central tendency and variability. For data that did not follow a normal distribution, the median and ranges were used. Statistical significance was defined as *p* < 0.05. To evaluate the relationships between all the measured parameters, Spearman correlation analysis was conducted. Spearman’s correlation coefficient (r) was used to assess the strength and direction of linear relationships between variables. Correlation values range from −1 to +1, where positive values indicate a direct correlation and negative values an inverse relationship. The absolute magnitude of the correlation coefficient indicates the strength of the relationship between two variables: 0.00–0.10 suggests a negligible correlation, 0.10–0.39 indicates a weak correlation, 0.40–0.69 suggests a moderate correlation, 0.70–0.89 indicates a strong correlation, while 0.90–1.00 signifies a very strong correlation [72].

## 5. Conclusions

The current study presents strong evidence connecting real-life exposure to mixtures of toxic metals with the disruption of pancreatic function. Differences related to sex, duration of exposure, and applied dosage were observed in almost all the measured parameters. Both male and female rats showed dose-dependent histopathological changes, with acinar cell vacuolization, vascular congestion, and alterations in Langerhans islet structure becoming more pronounced at higher doses and longer exposure durations. Male rats showed more pronounced vascular and Langerhans islet alterations, while female rats had more acinar cell change. Additionally, findings after 90 days indicated a progressive nature of these histopathological changes. The more pronounced decreases in glucose and amylase levels in males across various doses and exposure durations suggested that male rats are more affected by toxic metals than females. On the other hand, although MDH-1 activity also increased in males after prolonged exposure, females exhibited a more varied response depending on the treatment group. Toxic metal mixtures were also found to significantly disrupt oxidative stress parameters and mineral homeostasis, with male rats showing an adaptive response over time, while females continue to experience oxidative stress and protective adaptations. Our correlation analysis revealed that exposure to toxic metals might lead to co-accumulation patterns in pancreatic tissue, with significant interactions between toxic metals and bioelements indicating disruptions in enzyme function and mineral homeostasis, particularly in males. On the other hand, female rats showed lower initial correlations (28-day exposure) that intensified over time (90-day exposure). Further studies are needed to more precisely define and explain the possible causal relationship between real exposure to metal mixtures and the aetiology of this disease in humans.

## Figures and Tables

**Table 1:** Levels of toxic metals in the pancreas of male and female rats after 28/90 days of exposure to a mixture of toxic metals (As, Pb, Hg, Cd, Cr (VI), and Ni) at three/four different doses calculated based on a previous human biomonitoring study (M1/F1—doses based on the calculated lower confidence limit of the BMD for effects on hormone levels; M2/F2—doses based on the median concentrations of metals measured in the blood of the human population; M3/F3—doses based on the 95th percentile concentrations of metals measured in the blood of the human population; M4/F4: doses calculated based on the reference values from the existing literature). Statistical analysis: one-way ANOVA + LSD post hoc test (data presented as means ± standard deviations); Kruskal–Wallis test + Dunn’s post hoc test (data presented as medians with interquartile ranges). Significance levels are denoted as * *p* < 0.05; ** *p* < 0.01; *** *p* < 0.001 (vs. control group).

			Control	M1/F1	M2/F2	M3/F3	M4/F4
Male rats: 28 days of exposure	Pb (ng/kg)	Average	16	36 **	56 ***	43 ***	45 ***
		SD	3.3	13	2.8	14	11
	Cd (ng/kg)	Average	6.3	7.3	16 **	14 **	20 ***
		SD	1.8	2.4	8.5	2.2	3.4
	As (ng/kg)	25% Percentile	20	42	23	316 *	435 ***
		Median	24	66	27	372	447
		75% Percentile	27	72	39	415	467
	Hg (ng/kg)	Average	2.7	14	41 ***	49 ***	51 ***
		SD	0.34	1.5	9.3	7.4	17
	Cr (ng/kg)	25% Percentile	13	40	33	29	105
		Median	16	49	43	48	169 *
		75% Percentile	17	59	74	59	182
	Ni (ng/kg)	25% Percentile	102	165	182	155	228
		Median	112	254	290 *	178	263
		75% Percentile	116	411	314	245	285
			Control	F1	F2	F3	F4
Female rats: 28 days of exposure	Pb (ng/kg)	Average	29	37	40	41 *	25
		SD	10	8.5	9.2	12	4.7
	Cd (ng/kg)	Average	5.6	7.5	11 ***	11 ***	17 ***
		SD	0.70	1.3	1.2	2.7	1.4
	As (ng/kg)	25% Percentile	13	24	30	22	254
		Median	15	32	39	26	286 ***
		75% Percentile	18	53	42	31	458
	Hg (ng/kg)	25% Percentile	2.7	3.1	18	36	33
		Median	3.3	4.7	22	45 **	44 **
		75% Percentile	3.4	7.4	31	48	48
	Cr (ng/kg)	25% Percentile	25	42	35	41	134
		Median	27	52	51	42	139 **
		75% Percentile	29	65	87	72	143
	Ni (ng/kg)	25% Percentile	121	227	235	148	377
		Median	148	236	276	337	691 **
		75% Percentile	262	243	454	446	848
			Control	M1	M2	M3	M4
Male rats: 90 days of exposure	Pb (ng/kg)	Average	11	23 ***	19 **	25 ***	NA
		SD	1.3	5.3	2.2	3.9	NA
	Cd (ng/kg)	Average	2.4	3.3	3.4 *	3.7 **	NA
		SD	0.55	0.85	0.40	0.70	NA
	As (ng/kg)	25% Percentile	12	26	32	1006	NA
		Median	13	39	49	1274 ***	NA
		75% Percentile	16	53	69	1611	NA
	Hg (ng/kg)	Average	3.1	5.0	7.6 ***	13 ***	NA
		SD	0.76	1.9	1.7	2.1	NA
							NA
	Cr (ng/kg)	25% Percentile	20	23	23	23	NA
		Median	23	35	27	28	NA
		75% Percentile	25	45	43	31	NA
	Ni (ng/kg)	25% Percentile	55	144	126	142	NA
		Median	59	188	150	354 **	NA
		75% Percentile	65	235	313	495	NA
			Control	F1	F2	F3	F4
Female rats: 90 days of exposure	Pb (ng/kg)	Average	13	24 *	31 **	26 *	NA
		SD	3.7	11	9.2	3.8	NA
	Cd (ng/kg)	Average	2.8	4.7	6.3 **	7.0 ***	NA
		SD	0.30	2.0	1.4	2.1	NA
	As (ng/kg)	Average	17	55 ***	52 **	64 ***	NA
		SD	2.8	16	18	14	NA
	Hg (ng/kg)	25% Percentile	1.0	4.1	26	24	NA
		Median	3.0	4.2	30 *	45 **	NA
		75% Percentile	4.3	5.7	32	54	NA
	Cr (ng/kg)	Average	15	22 **	32 ***	33 ***	NA
		SD	2.5	2.7	5.3	2.8	NA
	Ni (ng/kg)	25% Percentile	76	147	160	143	NA
		Median	87	167	188 *	216 *	NA
		75% Percentile	94	209	225	315	NA

**Table 2:** Oxidative stress and antioxidant protection parameters in the pancreas of male and female rats after 28/90 days of exposure to a mixture of toxic metals (As, Pb, Hg, Cd, Cr (VI), and Ni) at three/four different doses calculated based on a previous human biomonitoring study (doses: M1/F1—calculated lower confidence limit of the Benchmark dose for effects on hormone levels; M2/F2—median concentration; M3/F3—95th percentile concentration; M4/F4—reference doses). Statistical analysis: one-way ANOVA + LSD post hoc test (data presented as means ± standard deviations); Kruskal–Wallis test + Dunn’s post hoc test (data presented as medians with interquartile ranges). Significance levels are denoted as * *p* < 0.05; *** *p* < 0.001 (vs. control group).

			Control	M1	M2	M3	M4
Male rats: 28 days of exposure	IMA (pg/g)	Average	31.31	25.46	33.90 *	24.73	45.33 ***
		SD	6.036	4.067	4.330	1.844	7.209
	MDA (μmol/g)	Average	40.32	25.95 *	36.28	37.30	36.82
		SD	7.443	7.663	9.134	9.779	6.864
	SH groups (mmol/g)	Average	0.088	0.422 ***	0.197	0.187	0.1714
		SD	0.0752	0.106	0.0450	0.0395	0.1403
	GSH (μmol/g)	Average	27.57	21.22	20.03	22.39	20.39
		SD	7.615	9.295	7.385	4.484	4.150
	SOD (IU/g)	25% Percentile	1.200	0.550	1.150	0.850	0.3500
		Median	1.700	1.050	1.300	1.500	0.7000
		75% Percentile	5.150	2.150	2.400	3.600	3.900
			Control	F1	F2	F3	F4
Female rats: 28 days of exposure	IMA (pg/g)	25% Percentile	24.24	22.02	24.96	27.13	30.26
		Median	28.32	28.30	29.81	29.20	34.83 *
		75% Percentile	30.23	34.47	34.54	31.83	48.72
	MDA (μmol/g)	Average	50.52	56.02	63.26 ***	58.04	41.54
		SD	11.05	6.176	4.274	13.03	5.160
	SH groups (mmol/g)	Average	0.138	0.119	0.0564 ***	0.153	0.1240
		SD	0.0225	0.0194	0.0145	0.00879	0.01281
	GSH (μmol/g)	Average	21.81	28.27	12.88 *	21.39	22.40
		SD	6.529	7.502	0.953	2.079	5.039
	SOD (IU/g)	25% Percentile	0.800	1.000	0.550	0.700	0.7000
		Median	1.200	1.200	1.200	1.000	1.000
		75% Percentile	1.400	1.600	2.200	3.700	2.000
			Control	M1	M2	M3	M4
Male rats: 90 days of exposure	IMA (pg/g)	25% Percentile	34.33	32.86	37.13	29.88	NA
		Median	35.08	38.85	41.69	36.04	NA
		75% Percentile	36.78	61.63	61.70	49.82	NA
	MDA (μmol/g)	Average	26.52	18.34	28.60	33.84	NA
		SD	9.941	5.699	10.86	6.146	NA
	SH groups (mmol/g)	25% Percentile	0.0680	0.107	0.0775	0.182	NA
		Median	0.107	0.193	0.195	0.234	NA
		75% Percentile	0.359	0.333	0.501	0.534	NA
	GSH (μmol/g)	25% Percentile	13.20	13.24	16.24	16.70	NA
		Median	25.68	14.11	23.45	29.17	NA
		75% Percentile	27.61	18.61	36.86	37.44	NA
	SOD (IU/g)	25% Percentile	1.400	1.550	1.500	2.500	NA
		Median	1.900	1.700	2.300	3.200	NA
		75% Percentile	3.050	2.700	3.550	4.100	NA
			Control	F1	F2	F3	F4
Female rats: 90 days of exposure	IMA (pg/g)	25% Percentile	31.85	37.18	32.47	34.03	NA
		Median	35.36	41.84 *	41.09	40.92	NA
		75% Percentile	37.43	48.61	41.61	56.08	NA
	MDA (μmol/g)	Average	19.84	23.82	24.94	26.56	NA
		SD	6.379	7.340	7.538	6.977	NA
	SH groups (mmol/g)	Average	0.0700	0.370 *	0.170	0.176	NA
		SD	0.0619	0.384	0.177	0.112	NA
	GSH (μmol/g)	Average	15.42	19.60	24.29 *	22.56	NA
		SD	4.468	6.999	8.404	5.256	NA
	SOD (IU/g)	25% Percentile	0.300	0.750	0.650	0.600	NA
		Median	0.500	0.900	1.400 *	1.600 *	NA
		75% Percentile	0.750	2.050	2.900	2.950	NA

IMA: ischemia-modified albumin; MDA: malondialdehyde; SH groups: sulfhydryl groups; GSH: glutathione; SOD: superoxide dismutase.

**Table 3:** Levels of bioelements in the pancreas of male and female rats after 28/90 days of exposure to a mixture of toxic metals (As, Pb, Hg, Cd, Cr (VI), and Ni) at three/four different doses calculated based on a previous human biomonitoring study (doses: M1/F1—calculated lower confidence limit of the Benchmark dose for effects on hormone levels; M2/F2—median concentration; M3/F3—95th percentile concentration; M4/F4—reference doses). Statistical analysis: one-way ANOVA + LSD post hoc test (data presented as means ± standard deviations); Kruskal–Wallis test + Dunn’s post hoc test (data presented as medians with interquartile ranges). Significance levels are denoted as * *p* < 0.05; ** *p* < 0.01; *** *p* < 0.001 (vs. control group).

			Control	M1	M2	M3	M4
Male rats: 28 days of exposure	Fe (μg/kg)	Average	31	27	21 **	16 ***	22 **
		SD	5.5	6.0	5.1	5.3	2.8
	Cu (μg/kg)	Average	1.6	1.0 ***	0.64 ***	0.75 ***	1.2 **
		SD	0.14	0.23	0.19	0.35	0.18
	Zn (μg/kg)	Average	20	13 ***	11 ***	12 ***	14 ***
		SD	2.2	2.9	2.3	3.2	2.0
	Mn (μg/kg)	Average	2.6	1.8 *	1.8 *	1.5 ***	2.1
		SD	0.084	0.42	0.77	0.56	0.22
	Ca (μg/kg)	25% Percentile	29	14	12	7.9	18
		Median	30	17 *	18 *	12 *	26
		75% Percentile	32	18	19	22	27
	Mg (μg/kg)	Average	222	269	239	197	391 *
		SD	66	68	160	105	34
			Control	F1	F2	F3	F4
Female rats: 28 days of exposure	Fe (μg/kg)	Average	23	29	31	25	28
		SD	8.4	12	12	8.4	9.2
	Cu (μg/kg)	Average	0.82	0.66	0.90	0.91	0.93
		SD	0.31	0.29	0.39	0.29	0.27
	Zn (μg/kg)	Average	15	15	18	19	17
		SD	2.1	3.7	5.1	5.3	3.1
	Mn (μg/kg)	Average	1.8	0.76 **	1.3	1.7	1.6
		SD	0.43	0.46	0.40	0.61	0.50
	Ca (μg/kg)	Average	20	15 *	16	17	17
		SD	0.79	2.3	4.2	5.0	5.1
	Mg (μg/kg)	Average	201	131	208	203	254
		SD	7.9	81	71	16	62
			Control	M1	M2	M3	M4
Male rats: 90 days of exposure	Fe (μg/kg)	25% Percentile	23	17	15	23	NA
		Median	30	21	16 *	25	NA
		75% Percentile	32	28	18	29	NA
	Cu (μg/kg)	Average	0.91	0.71 *	0.49 ***	0.75	NA
		SD	0.075	0.19	0.073	0.16	NA
	Zn (μg/kg)	Average	22	17 *	12 ***	17 *	NA
		SD	1.9	3.9	4.4	3.2	NA
	Mn (μg/kg)	Average	1.7	1.7	1.4	2.1	NA
		SD	0.61	0.55	0.36	0.31	NA
							NA
	Ca (μg/kg)	Average	23	22	14 ***	17 ***	NA
		SD	1.3	3.0	3.0	2.8	NA
	Mg (μg/kg)	Average	212	151 **	110 ***	117 ***	NA
		SD	9.0	24	4.1	4.8	NA
			Control	F1	F2	F3	F4
Female rats: 90 days of exposure	Fe (μg/kg)	Average	19	23	25	19	NA
		SD	3.0	9.7	13	5.7	NA
	Cu (μg/kg)	Average	0.97	0.69	0.90	0.66	NA
		SD	0.15	0.30	0.41	0.34	NA
	Zn (μg/kg)	Average	16	16	15	12 *	NA
		SD	1.7	3.1	3.0	5.0	NA
	Mn (μg/kg)	Average	1.6	1.5	1.7	1.5	NA
		SD	0.40	0.28	0.47	0.34	NA
	Ca (μg/kg)	Average	19	8.9 ***	9.2 ***	10 ***	NA
		SD	0.84	3.6	3.5	1.9	NA
	Mg (μg/kg)	Average	214	174 **	92 ***	73 ***	NA
		SD	6.1	23	23	16	NA

**Table 4:** Doses of toxic metals applied in the real-life simulation animal study, calculated based on a prior biomonitoring study.

		Cd (mg/kg b.w./day)	Pb (mg/kg b.w./day)	As (mg/kg b.w./day)	Hg (mg/kg b.w./day)	Cr (mg/kg b.w./day)	Ni (mg/kg b.w./day)
Male animals	M1(28)/M1(90)	2.333 × 10^−5^	2.120 × 10^−5^	0.006255316	8.085 × 10^−5^	1.264 × 10^−5^	0.0039306
	M2(28)/M2(90)	0.000126	0.000186666	0.004978616	0.004777846	0.000385713	0.0299077
	M3(28)/M3(90)	0.000366	0.00531664	0.089681714	0.0447651	0.003356639	0.1888047
	M4(28)	0.05	0.05	0.038	0.23	0.52	5
Female animals	F1(28)/F1(90)	3.50 × 10^−7^	0.000042971	0.005771	0.000579	4.5089 × 10^−6^	0.000161
	F2(28)/F2(90)	0.000214	0.00153903	0.068489	0.004033	0.00039499	0.041618
	F3(28)/F3(90)	0.000705	0.0095243	0.193642	0.036029	0.01673275	0.438417
	F4(28)	0.05	0.05	0.038	0.23	0.52	5

M1/F1—doses based on the calculated lower confidence limit of the Benchmark dose (BMD) for effects on hormone levels; M2/F2—doses based on the median concentrations of metals measured in the blood of the human population; M3/F3—doses based on the 95th percentile concentrations of metals measured in the blood of the human population; M4/F4: doses calculated based on the reference values from the existing literature; Cd: cadmium; Pb: lead; As: arsenic; Hg: mercury; Cr: chromium; Ni: nickel nickel (Ni).

**Table 5:** Methods for assessing oxidative stress and antioxidant defence parameters in rat pancreatic tissue following a 28- or 90-day oral exposure to toxic metal mixtures (Pb, Cd, As, Hg, Cr and Ni).

Parameter	Method Principle	Units	Reference
Malondialdehyde (MDA)	MDA levels are measured with thiobarbituric acid via absorbance after heating (100 °C, 5 min), cooling on ice (10 min), and centrifugation (10,000× *g*, at 4 °C, for 10 min).	µmol/mg protein	[67]
Ischemia-modified albumin (IMA)	Ischemia reduces albumin’s cobalt-binding ability at its amino terminal, measured colorimetrically with absorbance at 470 nm.	Absorbance units (ABSUs)	[68]
Total thiol group level (SH groups)	Absorbance of the yellow product from the reaction with 5,5′-dithiobis-(2-nitrobenzoic acid) (DTNB) in alkaline conditions (pH 9.0) is measured.	mmol/mg protein	[69]
Glutathione (GSH)	GSH is measured by reacting with DTNB in alkaline conditions (pH 9.0) after protein precipitation with 5% sulfosalicylic acid.	µmol/mg protein	[69]
Superoxide dismutase activity (SOD)	SOD activity is determined by its ability to inhibit adrenaline auto-oxidation at pH 10.2.	Percentage of the inhibition of adrenaline auto-oxidation (IU/mg protein)	[70]
Protein concentration	Coomassie Brilliant Blue G-250 reagent shifts absorbance from 465 to 595 nm upon binding to proteins, with bovine serum albumin as the standard.	mg	[71]

## Data Availability

Data will be made available on request.
